# Draft Genome Sequence of *Escherichia coli* K-12 (ATCC 10798)

**DOI:** 10.1128/genomeA.00573-17

**Published:** 2017-07-06

**Authors:** Daniela Dimitrova, Kathleen C. Engelbrecht, Catherine Putonti, David W. Koenig, Alan J. Wolfe

**Affiliations:** a Corporate Research and Engineering, Kimberly-Clark Corporation, Roswell, Georgia, USA; b Corporate Research and Engineering, Kimberly-Clark Corporation, Neenah, Wisconsin, USA; c Bioinformatics Program, Loyola University Chicago, Chicago, Illinois, USA; d Department of Biology, Loyola University Chicago, Chicago, Illinois, USA; e Department of Computer Science, Loyola University Chicago, Chicago, Illinois, USA; f Department of Microbiology and Immunology, Stritch School of Medicine, Health Sciences Division, Loyola University Chicago, Maywood, Illinois, USA

## Abstract

Here, we present the draft genome sequence of *Escherichia coli* ATCC 10798. *E. coli* ATCC 10798 is a K-12 strain, one of the most well-studied model microorganisms. The size of the genome was 4,685,496 bp, with a G+C content of 50.70%. This assembly consists of 62 contigs and the F plasmid.

## GENOME ANNOUNCEMENT

*Escherichia coli* is commonly found in the human intestinal tract, and while most strains are harmless members of the microflora, some are the causative agents of a variety of illnesses. Here, we announce the genome sequence of *E. coli* strain ATCC 10798 ([Migula] Castellani and Chalmers) isolated from the stool sample of a diphtheria convalescent patient in 1922 (1, 2). Per ATCC documentation, the genotype is λ^+^ F^+^ (https://www.atcc.org/products/all/10798).

For genome extraction and sequencing, the purchased culture isolate was grown on 5% sheep blood agar (BD BBL prepared plated medium) under 5% CO_2_ at 35°C for 48 h. To extract genomic DNA, cells were resuspended in 0.5 ml of DNA extraction buffer (20 mM Tris-HCl, 2 mM EDTA, 1.2% Triton X-100 [pH 8]), followed by the addition of 50 μl of lysozyme (20 mg/ml), 30 μl of mutanolysin, and 5 μl of RNase (10 mg/ml). After a 1-h incubation at 37°C, 80 μl of 10% SDS and 20 μl of proteinase K were added, followed by a 2-h incubation at 55°C. Two hundred ten microliters of 6 M NaCl and 700 μl of phenol-chloroform were added. After a 30-min incubation with rotation, the solutions were centrifuged at 13,500 rpm for 10 min, and the aqueous phase was extracted. An equivalent volume of isopropanol was added, and the solution was centrifuged at 13,500 rpm for 10 min after a 10-min incubation. The supernatant was decanted and the DNA pellet precipitated using 600 μl of 70% ethanol. Following ethanol evaporation, the DNA pellet was resuspended in Tris-EDTA and stored at −20°C.

Genomic DNA was diluted in water to a concentration of 0.2 ng/μl, as measured by a fluorometric-based method (Life Technologies, Inc.); 5 μl was used to obtain a total of 1 ng of input DNA. Library preparation of the isolated DNA was performed using the Nextera XT DNA library preparation kit. The library was sequenced on the MiSeq sequencer (Illumina) using the MiSeq reagent kit version 2 (500 cycles). The run produced 1,652,091 paired-end reads in total. BBDuk, part of the BBMap package (http://sourceforge.net/projects/bbmap/), was used to trim the reads. The trimmed reads were then assembled using SPAdes (version 3.5) (3), followed by scaffolding by SSPACE (4), which produced 63 contigs that varied in size from 680 bp to 507,483 bp (*N*_50_, 149,239 bp), with an average coverage of 156.4×. One of these contigs is a complete plasmid, a copy of the F plasmid. Furthermore, the *E. coli* strain sequenced is a lysogen; the sequence of the bacteriophage λ was identified within the genome assembly through BLAST. Annotations were produced using the software tool Peasant (5). Eight rRNA genes, 80 tRNA genes, and 4,519 protein-coding sequences were detected. Two confirmed clustered regularly interspaced short palindromic repeat (CRISPR) arrays were found (6). The genome size was 4,685,496 bp, with an observed G+C content of 50.70%.

### Accession number(s).

The draft whole-genome project for *E. coli* ATCC 10798 has been deposited at DDBJ/EMBL/GenBank under accession number NARG00000000. Raw sequence reads are deposited at DDBJ/EMBL/GenBank under accession number SRR5364300.
